# A comparative study between the Universal Spinal System^®^ (USS) and the CD Horizon^®^ Legacy™ (CDH) in the management of thoracolumbar fractures

**DOI:** 10.1051/sicotj/2019039

**Published:** 2019-11-29

**Authors:** Ahmed Samir Barakat, Ahmed Elattar, Khaled Fawaz, Ahmed Maher Sultan, Wael Koptan, Yasser ElMiligui, Abdelrazzaq Alobaid

**Affiliations:** 1 Orthopedics and Traumatology Department, Faculty of Medicine, Cairo University Kasr Al Ainy Street Cairo 11562 Egypt; 2 Orthopedic Department, Spine Surgery Unit, Al-Razi Hospital, Block 1 Jamal Abdul Nasser Street Kuwait City State of Kuwait

**Keywords:** Short-segment fixation, Pedicle screw, Polyaxial, Thoracolumbar burst fractures, USS Universal Spine System, CDH LEGACY

## Abstract

*Introduction:* For the treatment of unstable non-osteoporotic thoracolumbar fractures, the clinical and radiological outcome of short-segment fixation with the USS™ – Universal Spine System (DePuy Orthopedics, Inc., Warsaw, IN, USA) and the CD HORIZON^®^ LEGACY™ 5.5 Spinal System, (Medtronic Sofamor Danek USA, Inc., Memphis, TN, USA) were compared.

*Methods:* From March 2015 to January 2016, 40 consecutive patients with unstable traumatic thoracolumbar fractures who met our inclusion criteria were treated with either the USS system or CDH Legacy system. Segmental kyphosis angle (SKA) and anterior body height (ABH) of fractured vertebrae, and ASIA Impairment Scale (AIS) were evaluated. Radiological fusion was confirmed with plain X-rays and when indicated with computerized tomography (CT).

*Results:* The mean immediate kyphotic angle correction was 16.6° for the Schanz and 6.4 for the Legacy system, and the immediate mean anterior vertebral body height correction was 0.92 cm for the Schanz and 0.51 cm for the Legacy system. Our study shows a significant statistical difference between Schanz and Legacy systems regarding post-operative segmental kyphosis and height correction immediately postoperatively, at 6 months and at one-year follow-up (*p*-value < 0.005). The degree of pain reduction and neurological improvement was not influenced by the screw system.

*Conclusion:* Usage of USS in thoracolumbar fracture as a short-segment fixation led to a near anatomical reduction when compared to the Legacy system. However, there was no advantage regarding pain reduction and neurological outcome.

## Introduction

In the past decades, the demand for early mobilization and rapid return to work and activities of daily living has shifted the management clearly toward surgical intervention in the form of short-segment pedicular screw fixation. Advocators claim that non-surgical treatment was inferior in pain control, nonetheless significant functional outcome differences in physical function and quality of life were not reported [1]. Short-segment pedicular screw constructs for the treatment of thoracolumbar fractures gained popularity in the 1980s. This technique has many advantages as preserving motion segments, providing superior correction of kyphosis, and shorter operative time with reduced blood loss [2, 3].

In 1994, McCormack et al. [4] proposed the load-sharing classification (LSC) as a straightforward way to describe the extent of bony comminution, amount of fracture displacement, and amount of kyphotic deformity in a spinal fracture. Several years later, the classification proved to be valid and reliable. Accordingly, fractures with mild comminution (LSC ≤ 6) could be efficiently restored with short-segment pedicle instrumentation (four screws, two in each adjacent vertebra, skipping the fractured one). An anterior approach with anterior reconstruction has been recommended for highly comminuted fractures (LSC ≥ 7) [5]. Anterior column comminution was identified as an important predictor for mechanical failure and hence anterior procedures became more frequently performed in combination with posterior instrumentation [6].

To prevent this, various surgical techniques and instrumentations have been developed to restore or buttress the anterior column. The side loading Universal Spine System (USS) was developed in the early 1990s by the Technical Commission for Spinal Surgery of the Association for the Study of Internal Fixation (ASIF) in order to create a versatile spine fixation system capable of addressing many spinal pathologies effectively with a limited amount of instruments and fixation devices [7].

The top-loading Legacy system is one of the most versatile and frequently used contemporary pedicular screw systems reflecting the evolution of pedicular screws over the last four decades with a similar scope of indications as the USS. Per se the monoaxiality of the USS when compared to the polyaxiality of the Legacy system should provide a substantial mechanical advantage and the former could be regarded as one of the prototypes of monoaxial systems. This is still reflected by the fact that in fracture situations where a short-segment fixation is intended, monoaxial screws are still used by some authors [3].

The stimulus to compare the USS system versus one of the contemporary pedicular screw systems (Legacy) was the hypothesis that through the longer lever arm provided by the USS Schanz screws, a greater amount of correction could be achieved during the index operation provided that the patient has sufficient bone density to withstand the acute manipulation through the pedicels allowing for an almost anatomical correction. This study intends therefore to compare the clinical and radiological outcomes of the USS and Legacy systems to unveil the superiority of one system over the other in non-osteoporotic thoracolumbar fractures. To our knowledge, no study exists that would prove the theoretical superiority of the fixed angle device (USS) over a polyaxial top-loading system represented by the Legacy system in the treatment of thoracolumbar fractures, especially when considering that modern versatile screws like the Legacy system have largely substituted the elder systems and represent the key armamentarium in many clinics.

## Patients and methods

This prospective randomized comparative study involved 40 patients (36 males and 4 females) with a traumatic thoracolumbar fracture treated with short-segment posterior spinal fusion and transpedicular fixation.

This study was done at Al-Razi Hospital, Kuwait, State of Kuwait, during the period from March 2015 to January 2016. After Ethics Committee approval, all patients signed an informed and detailed consent describing the procedure, alternative treatment methods and possible complications.

All patients were examined clinically and radiologically on admission and assessed neurologically by the 2015 updated International Standards for Neurological Classification of Spinal Injury published by the American Spinal Injury Association (ASIA Impairment Scale = AIS) [8], Visual Analogue Score (VAS), spinal canal compromise and fracture instability. Postoperatively, the neurological status (AIS), VAS, and quality of fracture reduction were determined.

Radiological evaluation included measurements of both segmental kyphosis expressed in degrees and anterior vertebral height in centimeters (cm) and were quantified by Surgimap Spine for Windows (Nemaris Inc., New York, NY, USA).

All patients received a standardized preoperative workup including anteroposterior (AP) and lateral X-rays, computerized tomography (CT) scan, and magnetic resonance imaging (MRI) of the affected region. In case of polytrauma, a polytrauma spiral CT and further necessary radiological studies were requested. Patients with spinal cord injury were initiated on IV methylprednisolone 30 mg/kg/h for 1 h as a bolus dose followed by 5.4 mg/kg/h for the next 23 h. Bed rest was initiated when there was mechanical or neurological instability and all patients received routinely enoxaparine 40 mg SC once daily and NSAIDs and/or opiate derivates for pain control.

Inclusion criteria were:

- mechanically unstable thoracolumbar fractures with loss of vertebral height > 50%, kyphotic angle > 20%, and canal compromise > 20%;
- associated neurology;
- skeletally mature patient (15–60 years).

Exclusion criteria were:

- active systemic infection;
- osteoporosis;
- sensitivity to implant material;
- being medically unfit for anesthesia.

To randomly distribute the patients into Group 1 (USS) or Group 2 (Legacy), computerized randomization was done ahead of the study with Research Randomizer (Version 4.0) from https://www.randomizer.com so that each group included 20 patients. Statistical analysis was done with IBM SPSS™ version 18.0 (IBM Corp., Armonk, NY, USA). In addition to the standard descriptive statistical analyses, the chi-square for categorical, and Student’s *t*-test for nominal data were employed. Throughout the study, the significance level and the confidence interval were set to *p* = 0.05 and CI = 95%, respectively.

Patients with isolated thoracolumbar fracture were discharged during the first 2 weeks from admission with a mean post-operative hospital stay of 10.5 days (range: 5–14 days). This time was extended in patients with neurological deficits or those with other compounding osseous injuries.

Routine clinical and radiological evaluation was done at 6 months and 12 months after discharge.

### Operative technique

#### Universal Spinal System (USS)

After receiving general anesthesia and 2 g ceftriaxone, IV patients were placed onto a fluoroscopic imaging table in the prone position on adequately sized and strategically positioned frame pollsters to obtain ligamentotaxis at the involved segments. Fluoroscopic evaluation of the spinal column was performed to assess reduction which was applied carefully through lumbar extension and when there was no retro-pulsed fragment through opposing longitudinal traction applied through the wrist and ankles. Appropriate skin markings were applied to assist in determining the level of surgical exposure which was achieved through a routine midline approach.

The corresponding pedicle entry points were identified using anatomical landmarks and a blunt K-wire was placed and a fluoroscopic image was obtained in the posterior-anterior and lateral projections to confirm localization of the pedicle. A pedicle awl was then placed into the pilot hole and advanced slowly down the pedicle approximately 15–20 mm in depth, with 10–15° medial inclination and an appropriate degree of cephalad-caudad inclination.

A Schanz screw was inserted into the pedicle under lateral radiographic imaging. The tips of the screw should end about 1 cm short of the anterior vertebral cortex.

The appropriate rod length was selected, allowing for any necessary distraction and was then assembled through the low-profile clamps and guided down to the Schanz screws.

*Sagittal alignment correction*:

- Intact posterior wall: the cannulated socket wrenches were placed bilaterally over the most cranial Schanz screws and the clamps were tilted caudally to lordose the spine. The desired angulation was secured by tightening the nut on the clamp with the socket wrench. Analogously, the step was repeated with caudal screws which were tilted cranially. Radiographic imaging was used to confirm vertebral height and screw positioning.
- Fractured posterior wall: If retro-pulsed fragments are in the intraspinal canal and the posterior longitudinal ligament is intact, distraction can be performed with the socket wrenches by ligamentotaxis. A half ring was used during sagittal alignment correction to protect the posterior aspect of the vertebral body during manipulations acting as a stop. Approximately 10° of correction could be achieved, if the half rings were placed 5 mm from the clamp. Finally, all connectors were tightened then the shafts of the Schanz screws were cut off. The low-profile clamp was used both cranially and caudally.

#### CD Horizon Legacy Spinal System

The entry point and trajectory of the CD screws was determined with the implant specific instruments similarly to the USS screws as described above.

After establishing the proper screw hole, a screw was spanned onto either the fixed angle or multiaxial screwdriver and slowly advanced down the pedicle to ensure proper tracking, while allowing for viscoelastic expansion, then, checked radiographically at this time to ensure intraosseous screw placement. The rods were applied as recommended by the manufacturer and careful reduction was attempted by applying distraction through the distraction instrument applied to the adjacent pedicular screws unilaterally over the rods which were tightened, and the final reduction was assessed by image intensifier.

Mobilization in the form of full weight-bearing was initiated in the neurologically intact patients on the first post-operative day and suction drains were removed on the second post-operative day. Patients were advised to avoid excessive trunk flexion extension and rotational movements for the next 3 months and return to full pre-traumatic activity after 6 months, postoperatively. This is to prevent overloading of the pedicular screws itself and to preserve the screw bone interface, thus preventing loosening of the pedicular screws.

## Results

**Figure 1:**
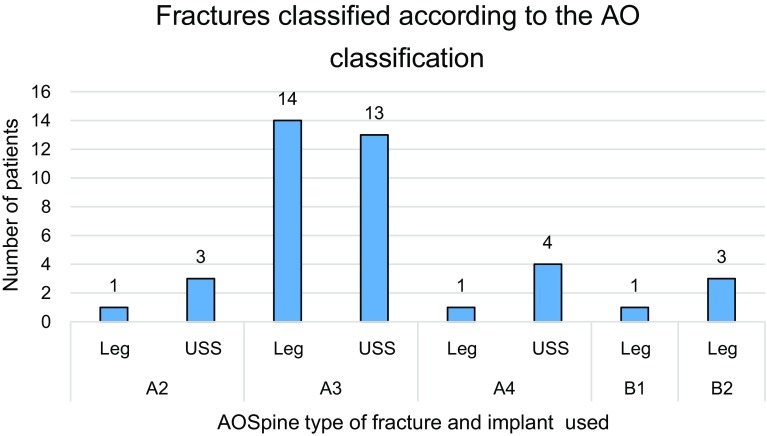
Number of patients according to AOSpine Classification and implant used.

The fracture type according to the AOSpine thoracolumbar spine injury classification system [9] and the selected fixation implants are depicted in Figure 1. The most common fracture type treated was A3 and included 27 patients (14 in the Legacy and 13 in the USS group). No statistically significant difference regarding the mean injury-surgery interval could be verified which was 2.4 days (0–7 days) and 2.25 days (0–5 days) for the USS and Legacy group, respectively. Moreover, the injury-surgery interval had no effect on the functional or radiological outcome in both groups.

Both the USS and Legacy group showed a normal distribution of the preoperative anterior vertebral height and kyphosis angle as shown by the Shapiro-Wilk test with a *p*-value of 0.22 and 0.23 and of 0.184 and 0.326, respectively.

Post-operative anterior vertebral heights and kyphosis angles, immediately postoperatively, at 6 months, and at 12 months were significantly better in the USS group with *p* values ranging from *p* = 0.04 to *p* < 0.001 (Table 1).

**Table 1:** Statistical significance of USS vs. Legacy System regarding angular kyphosis and anterior vertebral height.

	Independent samples *t*-test of USS vs. Legacy system				
	*t*	df	*p*	Mean difference	SE difference
Preop kyphosis	0.735	38.00	0.467	2.750	3.741
Post-op kyphosis	−2.206	38.00	0.034	−7.450	3.378
6 months kyphosis	−2.088	38.00	0.044	−7.205	3.450
12 months kyphosis	−2.192	38.00	0.035	−7.592	3.464
Preop height	0.293	38.00	0.771	0.044	0.152
Post-op height	−2.919	38.00	0.006	−0.360	0.123
6 months height	−3.009	38.00	0.005	−0.372	0.124
12 months height	−4.615	38.00	<.001	−0.501	0.109

*Note.* Student’s *t*-test.

The average anterior vertebral height and kyphosis angle for the CD Legacy system at 12 months was 2.00 cm and −7.39°, respectively, and for the USS 2.41 cm and 0.2°, respectively.

VAS score improved from severe pain (mean = 8, range: 7–10) to mild-to-moderate pain (mean = 3, range: 2–4) in 37 patients. Three patients reported no pain at 12-month follow-up with no statistical difference between USS and Legacy groups.

**Figure 2:**
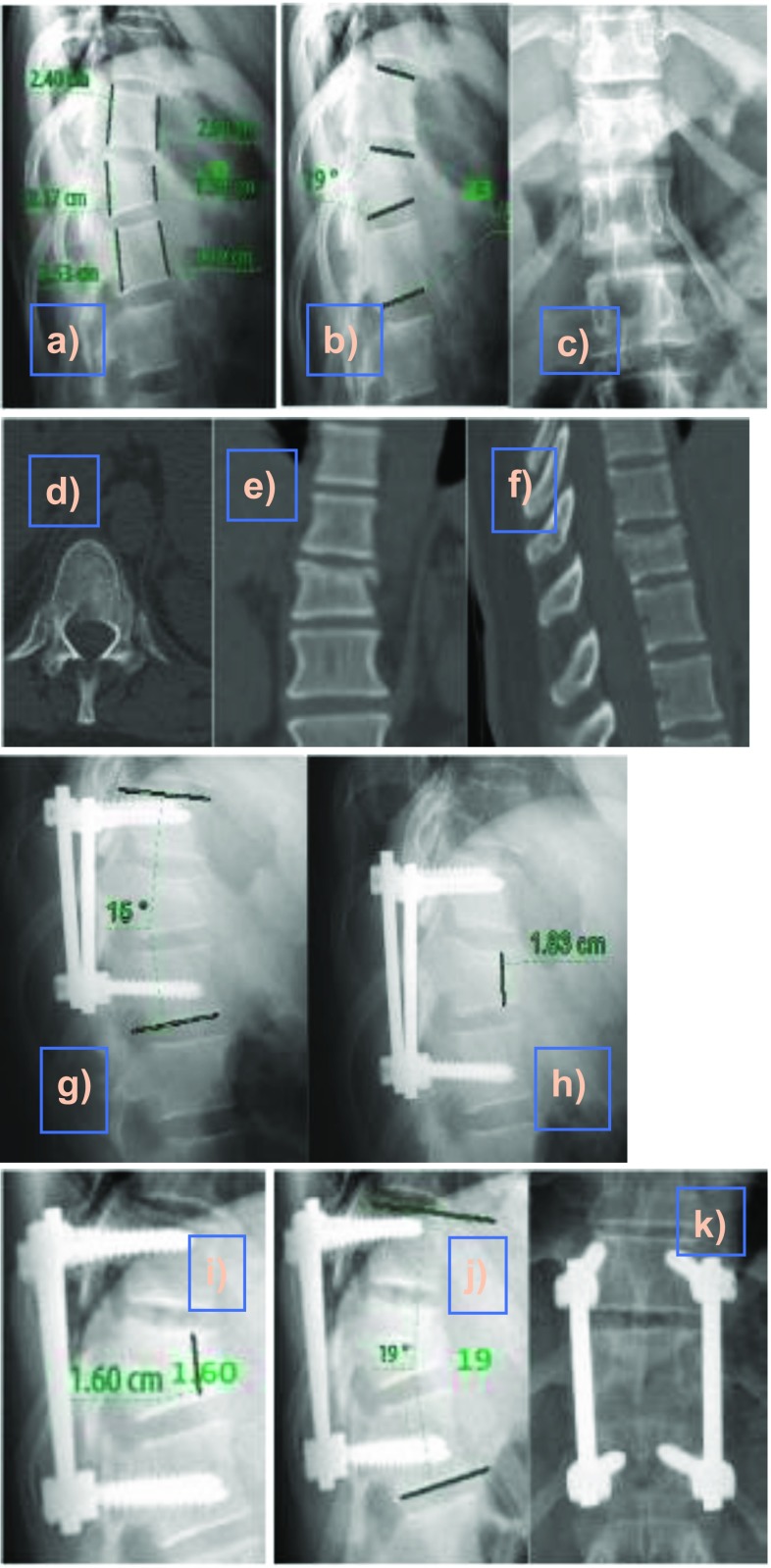
D11 fracture after fall from a height in a 35-year-old male without neurological deficit. (a–c) Preoperative measurements of angular kyphosis and anterior vertebral height; (d–f) preoperative CT; (g, h) immediately post-operative lateral X-rays after application of a Legacy System, and (i–k) follow-up AP and lateral X-rays at 12 months.

**Figure 3:**
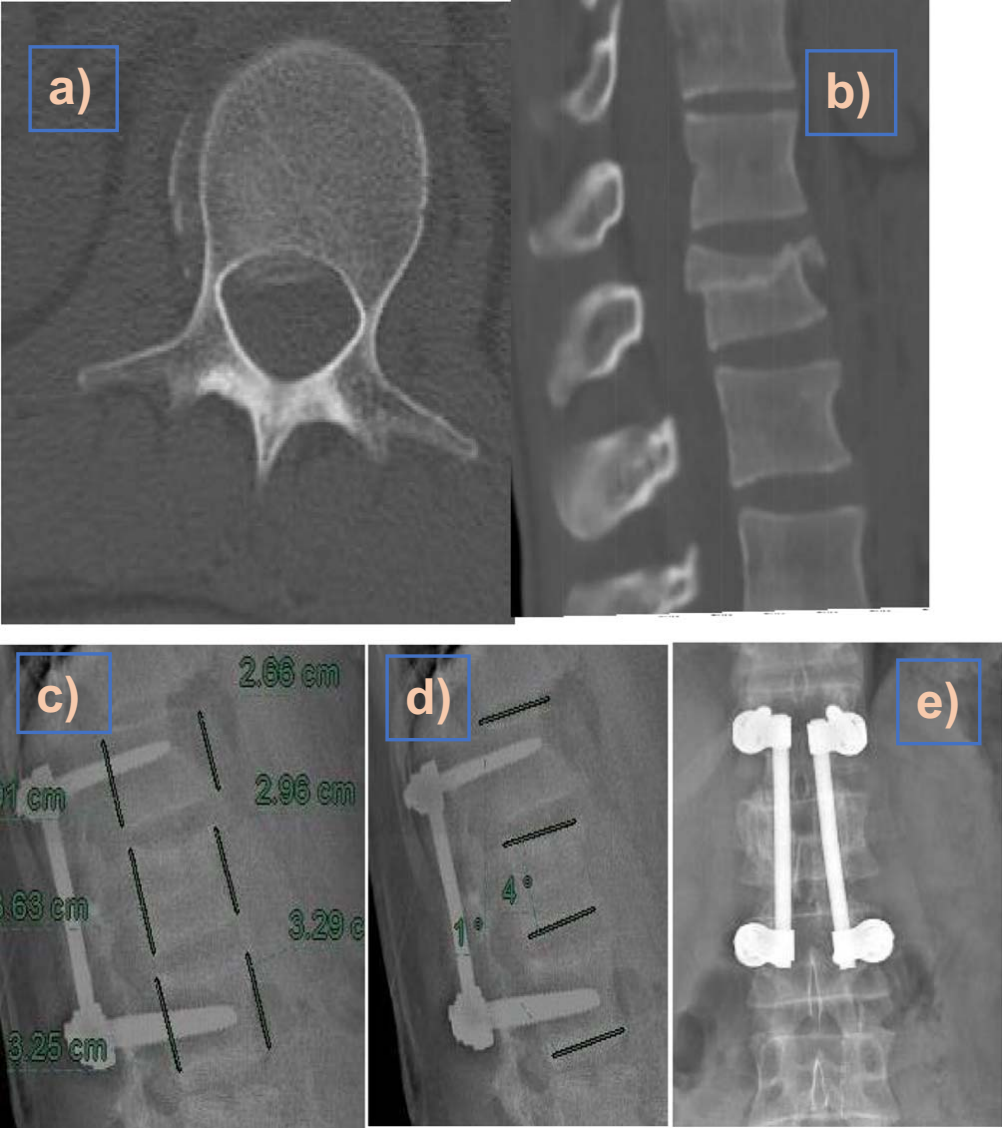
36-year-old male patient after RTA with a burst fracture of L1 (AO type A3) and associated bilateral calcaneal fracture with Frankle grade E. Patient was operated on the fifth day after trauma using USS. (a, b) Axial and sagittal CT scan delineating the burst fracture at L1; (c–e) lateral and AP X-rays after one-year follow-up showing good preservation of the vertebral height and sagittal angular alignment.

Regarding operative and radiation exposure time, there were no significant differences between the two groups with operative time ranging from 60 min to 125 min (mean = 91.8 min) and radiation exposure time ranged from 8 s to 40 s (mean = 20.6 s) (Figures 2 and 3).

### Post-operative neurological assessment

None of the three patients presenting initially with complete paralysis showed neurological improvement. Neurological improvement was observed in four patients with ASIA C to improve to ASIA E at final follow-up. Another two patients with ASIA grade C improved one grade only to be ASIA D at final follow-up. Two patients with initial ASIA grade D improved to ASIA grade E. In the remaining patients, no post-operative neurological deterioration was recorded. Of the eight patients with partial neurological deficit, six patients were operated during the first 24 h after trauma and the other two patients were operated on the second and third days, respectively. There was no statistically significant difference between the neurologically intact patients and patients with neurological deficit regarding correction of deformity and spinal canal decompression.

Most of the patients with initial partial neurological deficit (ASIA grade C and D) were able to return to their previous activity levels. A statistical difference between USS and CDH systems at the final follow-up regarding the neurological status could not be identified.

### Postoperative complications

Two patients, both in the USS group, developed mild superficial wound infection and were treated with systemic antibiotics and regular dressing.

One case in the Legacy group developed a deep wound infection (*Staph. aureus*) and was successfully treated by wound debridement and culture sensitive systemic antibiotics (clindamycin 600 mg IV t.i.d. for 1 week then orally for another 4 weeks).

One patient with complete paraplegia developed deep venous thrombosis on the fourth post-operative day and was treated by a therapeutic doses of enoxaparin SC (80 mg, bds).

Three patients developed urinary tract infection (two patients with complete paraplegia Frankel grade A and one patient with Frankel grade C) and were treated with antibiotics according to culture and sensitivity.

On the first post-operative day, one patient showed a transient paralytic ileus which was conservatively treated with a nasogastric tube for 48 h.

After 8 months of fixation, one patient in the Legacy group presented with a fracture of the right inferior pedicular screw due to de novo back trauma. Although there was mild back pain without any neurological symptoms or acute vertebral fracture, he was treated conservatively due to the sufficient bony healing with back support and pain medication.

## Discussion

Compared with conventional long-segment fixation, short-segment constructs provide the advantages of preserving motion segments and requiring smaller exposure with less spinal muscle damage [10].

In 2010, Gelb et al. [11] concluded that short-segment pedicle screw instrumentation is an effective technique to reduce and stabilize thoracic and lumbar spine fractures even in highly comminuted vertebral fractures obviating anterior column reconstruction.

Parker et al. [12] in 2000 concluded that thoracolumbar fracture can be stabilized with short-segment fixation with no implant failures and an average loss of correction of 4°.

Korovessis et al. [13] in 2006 conducted a prospective series of 40 consecutive patients with acute lumbar burst fractures treated with combined one-stage anterior stabilization with mesh cage and minimally invasive short-segment transpedicular fixation (SSTF) and fusion alone or SSTF alone encompassing the fractured vertebrae in both the study groups. The average length of follow-up was 46 and 48 months, respectively. A significant loss (5°) of correction in post-traumatic kyphosis was observed only in the short-segment fixation group, while spinal canal clearance was similar after surgery in both groups. No instrumentation failure or significant loss of sagittal alignment occurred; however, the authors do not recommend SSTF in type A3 fractures in the mid-lumbar spine. Similarly, we report only one implant failure without mechanical consequence and both implant systems were used in A3 and A4 burst fractures. We did not apply pedicular screws to the fractured vertebrae and average kyphosis angle at 12 months was −7.39° and 0.2° for CD legacy and USS, respectively. Analogous, the average anterior vertebral height for the CD Legacy system at 12 months was 2.00 cm and for the USS 2.41 cm.

Our planned follow-up was admittedly only 12 months, but we believe that at this point no further bony settling is to be expected due to completed osseous union. Nevertheless, the clinical long-term follow-up cannot be foreseen by the underlying study.

Post-surgical kyphosis may be secondary to screw bending, further osseous collapse, or vertebral translation without hardware failure. Recurrent kyphosis after posterior reduction also appears to be a result of creeping of the nucleus pulposus back into the depressed central area [14]. Posterior reduction probably reduces only the periphery of the endplate with strong annular attachments, while the central areas remain depressed.

The long- and mid-term effects of a compromised post-traumatic sagittal profile with regard to adjacent disc decompensations and degenerative changes of the spine remain a debatable area for further research [1, 15].

Regardless of the pedicular screw type used, there was a marked reduction of acute pain reflected by the VAS, which decreased to an average of 3 (range: 2–4) when compared to the preoperative status. Different screw types did also not affect the neurological outcome of the patients of this series. Similar findings were reported by other authors [16, 17].

In this study, we used an open approach for both implant systems; however, a plethora of reports describe good to excellent results with percutaneous fixation systems [18–20]. This could serve as a stimulus to compare our results with contemporary percutaneous systems and even develop a percutaneous version of the USS to benefit from its increased lever arm in addition to the obvious advantages of the minimal invasive approach. Especially in the polytrauma setting and with consideration to orthopedic damage control, a percutaneous technique might prove favorable to open approaches [21]. No intended fusion was performed in the underlying study and Elkhateeb and Mahmoud found that there was no difference if the dorsolumbar fracture had a load sharing score < 7 [22]. Short-segment fixation with intermediate pedicular screw application into the fractured vertebrae unilaterally or bilaterally was shown to have favorable biomechanical characteristics as reduced von Mises stress and lower intradiscal pressure in the adjacent segments and lower occurrence of adjacent segment degeneration, especially when using a polyaxial screw system [23]. Hence many surgeons treating thoracolumbar fractures clearly shifted their practice to short-segment fixation with intermediate fixation [3].

Usage of USS in thoracolumbar fracture as a short-segment fixation led to a sustained near anatomical reduction when compared to the Legacy system. However, there was no advantage regarding pain reduction and neurological outcome.

## Conflict of interest

The authors certify that they have no financial conflict of interest (e.g., consultancies, stock ownership, equity interest, patent/licensing arrangements, etc.) in connection with this article.

## Funding

The authors declare that they did not receive any public or private funding, whatsoever.
